# L,L-diaminopimelate aminotransferase (DapL): a putative target for the development of narrow-spectrum antibacterial compounds

**DOI:** 10.3389/fmicb.2014.00509

**Published:** 2014-09-26

**Authors:** Alexander J. Triassi, Matthew S. Wheatley, Michael A. Savka, Han Ming Gan, Renwick C. J. Dobson, André O. Hudson

**Affiliations:** ^1^Thomas H. Gosnell School of Life Sciences, Rochester Institute of TechnologyRochester, NY, USA; ^2^School of Science, Monash University MalaysiaBandar Sunway, Malaysia; ^3^Department of Biochemistry and Molecular Biology, Bio21 Molecular Science and Biotechnology Institute, The University of MelbourneParkville, VIC, Australia; ^4^Biomolecular Interaction Centre, School of Biological Sciences, University of CanterburyChristchurch, New Zealand

**Keywords:** amino acid, antibacterial, antibiotic, lysine, pyridoxal-5′-phosphate, diaminopimelate, L,L-diaminopimelate aminotransferase, peptidoglycan

## Abstract

Despite the urgent need for sustained development of novel antibacterial compounds to combat the drastic rise in antibiotic resistant and emerging bacterial infections, only a few clinically relevant antibacterial drugs have been recently developed. One of the bottlenecks impeding the development of novel antibacterial compounds is the identification of new enzymatic targets. The nutritionally essential amino acid anabolic pathways, for example lysine biosynthesis, provide an opportunity to explore the development of antibacterial compounds, since human genomes do not possess the genes necessary to synthesize these amino acids *de novo*. The diaminopimelate (DAP)/lysine (lys) anabolic pathways are attractive targets for antibacterial development since the penultimate lys precursor *meso*-DAP (*m*-DAP) is a cross-linking amino acid in the peptidoglycan (PG) cell wall of most Gram-negative bacteria and lys plays a similar role in the PG of most Gram-positive bacteria, in addition to its role as one of the 20 proteogenic amino acids. The L,L-diaminopimelate aminotransferase (DapL) pathway was recently identified as a novel variant of the DAP/lys anabolic pathways. The DapL pathway has been identified in the pathogenic bacteria belonging to the genus; *Chlamydia*, *Leptospira*, and *Treponema*. The dapL gene has been identified in the genomes of 381 or approximately 13% of the 2771 bacteria that have been sequenced, annotated and reposited in the NCBI database, as of May 23, 2014. The narrow distribution of the DapL pathway in the bacterial domain provides an opportunity for the development and or discovery of narrow spectrum antibacterial compounds.

## Introduction

The rise in the number of multidrug-resistant bacteria has led to a significant increase in the morbidity and mortality of humans infected with pathogenic bacteria. As such, the development and/or discovery of novel antibacterial compounds critical for the improvement of human health are necessary to combat current and emerging bacterial infections and/or diseases. Antibacterial agents generally fall into two classes of compounds; bactericidal and bacteriostatic. Bactericidal means that the compound is able to kill the bacteria while bacteriostatic means that the agent is able to prevent growth of the bacteria (Pankey and Sabath, 2004).

Of the 20 common proteogenic amino acids, nine are considered nutritionally essential due to the fact that these nine amino acids cannot be made by animals, particularly humans. As such, these amino acids are deemed indispensable for growth and development due to their overarching role in protein synthesis, in addition to their roles in other pathways, and must be acquired through dietary means. The amino acids that are deemed essential are leucine, isoleucine, valine, threonine, methionine, tryptophan, phenylalanine, histidine, and lysine. Of the nine, lysine is of interest since the pathway is also important for the synthesis of the peptidoglycan (PG) cell wall in bacteria.

**Figure 1:**
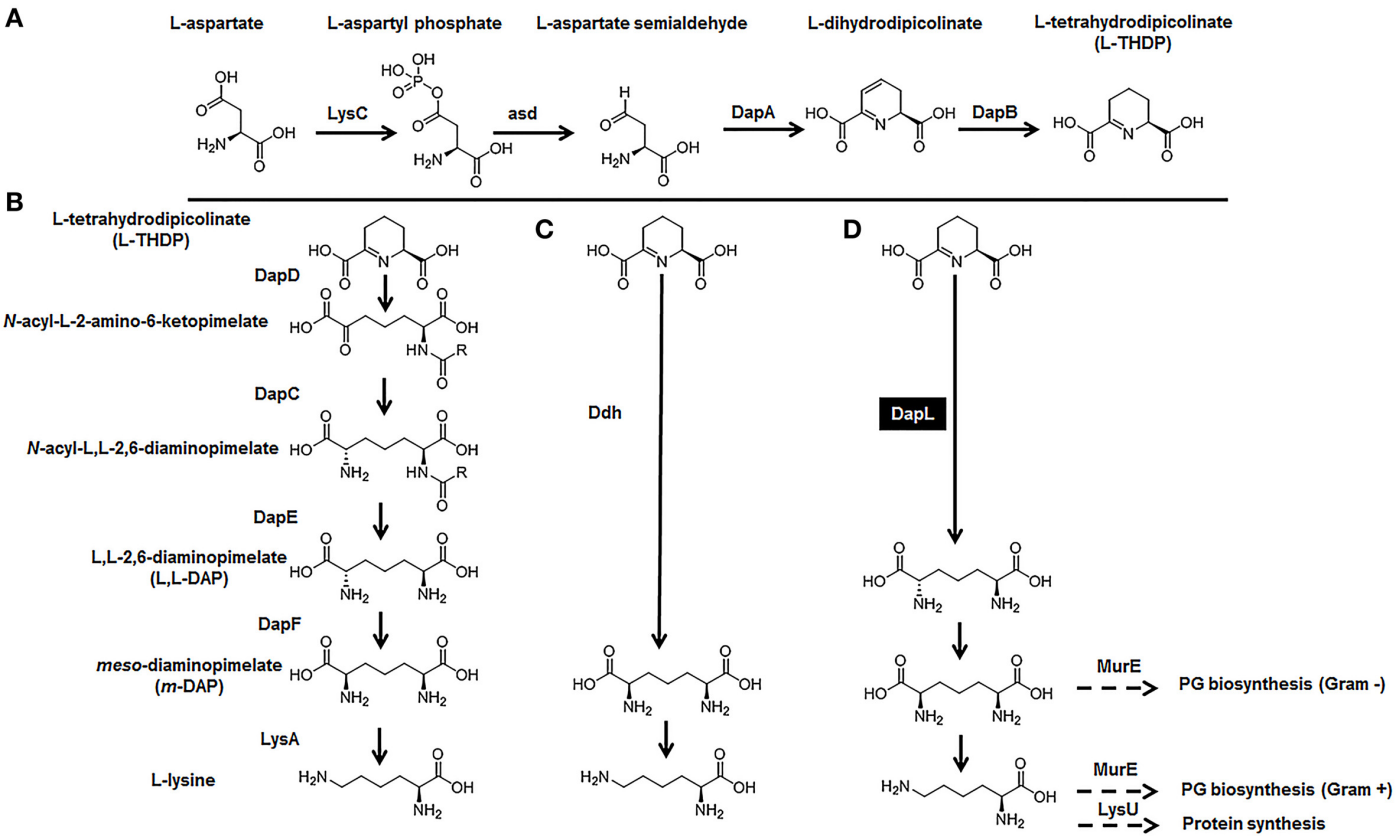
**The DAP/lys anabolic pathways. (A)** The synthesis of THDP from aspartate, **(B)** the acyl pathways, **(C)** the Ddh pathway, and **(D)** the DapL pathway. The abbreviations of the enzymes in addition to the enzymes reaction definitions are listed in Table 1.

In nature, the essential amino acid lysine (lys) is synthesized by certain organisms utilizing one of two pathways. The α-aminoadipic acid (AAA) pathway is predominantly employed by fungi and is present in a few species belonging to the domain archaea (Nishida et al., 1999; Velasco et al., 2002). Photosynthetic organisms and most bacteria employ the diaminopimelate (DAP) pathway. To date, four variants of the DAP/lys anabolic pathways has been elucidated and characterized. The acyl pathways, which utilize succinylated (succinyl-CoA) or acetylated (acetyl-CoA) intermediates, is present in most bacterial species; the *meso*-diaminopimelate (*m*-DAP) dehydrogenase (Ddh) pathway, which was initially discovered in *Bacillus sphaericus*, *Corynebacterium glutamicum*, and *Brevibacterium* sp. (Misono et al., 1976, 1979a,b, 1986; White, 1983), and the recently discovered L,L-diaminopimelate (L,L-DAP) aminotransferase (DapL) variant pathway (Hudson et al., 2006, 2008; McCoy et al., 2006) (Figure 1). The synthesis of lysine *de novo* from the DAP pathways can be divided into three main steps. The first step characterized by the synthesis of tetrahydrodipicolinate acid (THDP) from aspartate (asp) is a general feature of all four variants. This conversion is carried out in a series of reactions by the enzymes; LysC (EC: 2.7.2.4), asd (EC: 1.2.1.11), DapA (EC: 4.2.1.52), and DapB (EC: 1.2.1.26) (Figure 1A). The second step, which constitutes the conversion of THDPA to the penultimate intermediate *m*-DAP, defines the uniqueness of the DAP variant pathways. In the acyl pathways, four enzymes needed for the conversion of THDPA to *m*-DAP. These reactions are carried out by the enzymes DapD (EC: 2.3.1.117), DapC (EC: 2.6.1.17), DapE (EC: 3.5.1.18), and DapF (EC: 3.5.1.18) (Figure 1B). In the diaminopimelate dehydrogenase (Ddh) (EC:1.4.1.16) pathway, *m*-DAP is synthetized from THDP in a single reaction which is capable of circumventing the DapD, DapC, DapE, and DapF enzymatic steps that are present in the acyl pathways (Figure 1B). In the L,L-diaminopimelate aminotransferase (DapL) pathway, L,L-DAP is synthesize from THDP in a single transamination reaction bypassing the DapD, DapC and DapE steps that are present in acyl DAP pathways (Figure 1B). The conversion of *m*-DAP to lysine via a decarboxylation reaction facilitated by *m*-DAP decarboxylase (LysA EC: 4.1.1.20) defines the ultimate step in the anabolism of lysine and it is a common feature of the DAP/lys anabolic variants (Figure 1). The reaction definition of each enzyme involved in the DAP/lys biosynthesis pathway is presented in Table 1.

**Table 1:** **List of DAP/Lys anabolic genes and reaction definitions**.

**Symbol**	**Gene name**	**Reaction definition**
LysC	Aspartate kinase	ATP + L-aspartate <=> ADP + 4-phospho-L-aspartate
asd	Aspartate semialdehyde dehydrogenase	L-aspartate 4-semialdehyde + orthophosphate +NADP^+^ <=> 4-phospho-*L*-aspartate + NADPH + H^+^
DapA	Dihydrodipicolinate synthase	L-aspartate 4-semialdehyde + Pyruvate <=> L-2,3-dihydrodipicolinate + 2H_2_O
DapB	Dihydrodipicolinate reductase	2,3,4,5-tetrahydrodipicolinate + NADP+ <=> L-2,3-dihydrodipicolinate + NADPH + H^+^
DapD	2,3,4,5-tetrahydropyridine-2,6-dicarboxylate *N*-acyl-transferase	Acyl-CoA + 2,3,4,5-tetrahydrodipicolinate + H_2_O <=> CoA + *N*-acyl-2-L-amino-6-oxoheptanedioate
DapC	Acyl-diaminopimelate aminotransferase	*N*-acyl-L,L-2,6-diaminoheptanedioate + 2-oxoglutarate <=> *N*-acyl-2-L-amino-6-oxoheptanedioate + L-glutamate
DapE	Acyl-diaminopimelate deacylase	*N*-acyl-L,L-2,6-diaminoheptanedioate + H_2_O <=> acyl + L,L-2,6-diaminoheptanedioate
Ddh	Diaminopimelate dehydrogenase	*meso*-2,6-diaminoheptanedioate + NADP^+^ + H_2_O <=> L-2-amino-6-oxoheptanedioate + NH_3_ + NADPH
DapL	L,L-diaminopimelate aminotransferase	L,L-2,6-diaminopimelate + 2-oxoglutarate <=> 2,3,4,5-tetrahydrodipicolinate + L-glutamate +H_2_O
DapF	Diaminopimelate epimerase	L,L-2,6-diaminopimelate <=> *meso*-2,6-diaminopimelate
LysA	*meso*-diaminopimelate decarboxylase	*meso*-2,6-diaminopimelate => L-lysine + CO_2_
MurE	UDP-*N*-acetylmuramoylalanyl-D-glutamate–2, 6-diaminopimelate ligase	UDP-*N*-acetylmuramoyl-L-alanyl-D-glutamyl-meso-2,6-diaminopimelate:D-alanyl-D-alanine ligase+ ADP
LysU	Lysine -tRNA synthetase	ATP + lysine + tRNA(lys) <=> AMP + Diphosphate + L-lysyl-tR

*The list was generated using the genomic information deposited in the Integrated Microbial Genomes (IMG) database (http://img.jgi.doe.gov/cgi-bin/w/main.cgi)*.

**Figure 2:**
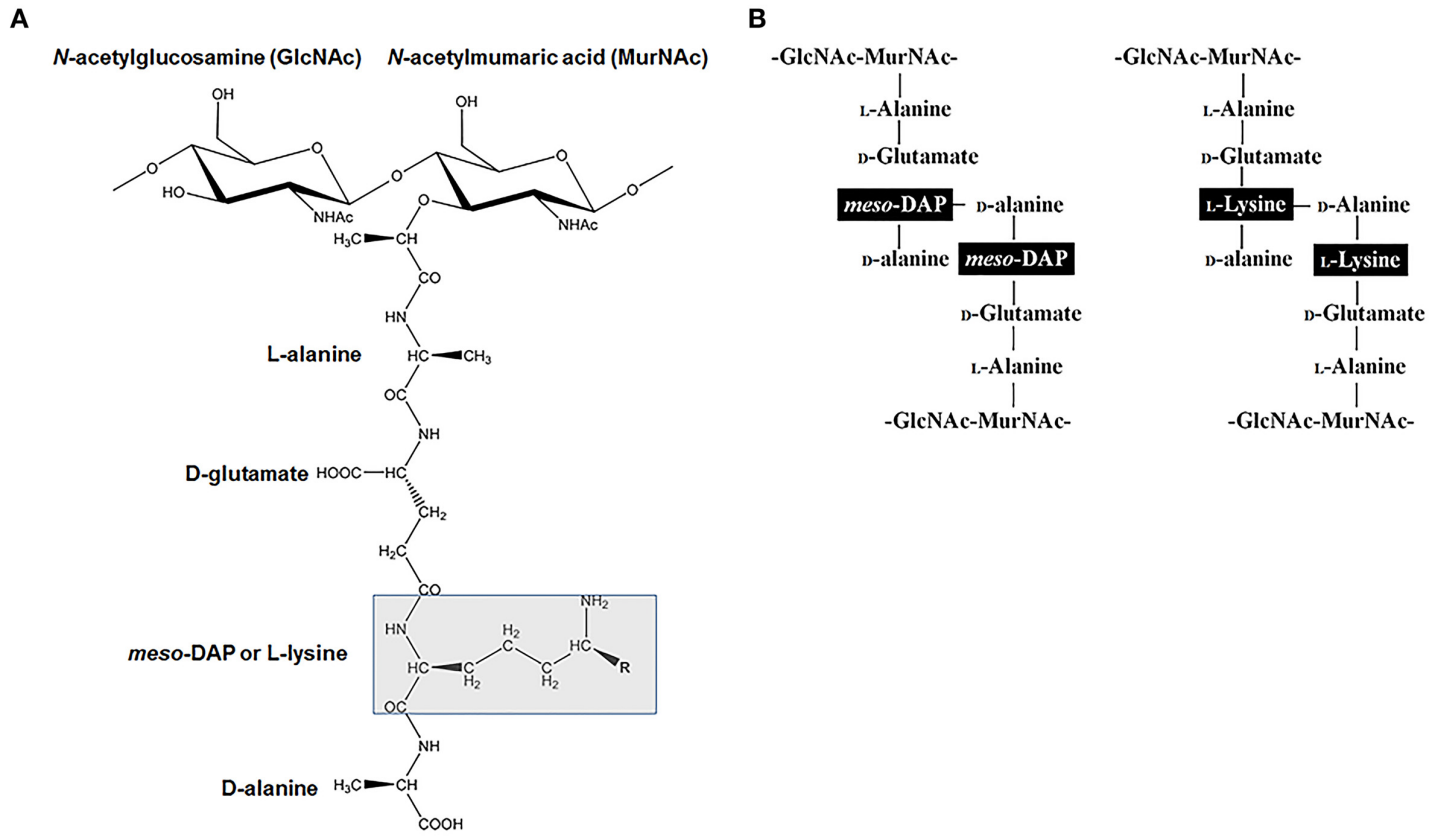
**The involvement of *m*-DAP and lys in the structure of peptidoglycan (PG) structure. (A)** The monomeric unit of PG showing the disaccharide of GlcNAc and MurNAc. The amino acid at the highlighted third position is *m*-DAP in most Gram negative bacteria and lysine in most Gram positive bacteria. **(B)** Schematic showing PG crossing linking with *m*-DAP to D-alanine in most Gram negative bacteria and lysine to D-alanine in most Gram positive bacteria.

In addition to making lys for protein synthesis, the DAP/lys pathways is also vital for the synthesis of the PG cell wall in most bacteria. The bacterial cell wall has an important role in buffering the internal and external forces. The cell wall is predominantly composed of a cross-linked polymeric layer of β-1,4-linked disaccharide of *N*-acetylglucosamine (GlcNAc) and *N*-acetylmumaric acid (MurNAc) that is connected to a peptide stem. The peptide stem is connected to MurNAc with the general sequence of L-alanine-D-glutamate-X-D-alanine where the third amino acid denoted by X is either *m*-DAP or lys (Figure 2A). *m*-DAP is the cross linking amino acid in the cell wall of most Gram-negative bacteria and lys serve a similar role in Gram-positive bacteria (Hutton et al., 2007) (Figure 2B). The addition of *m*-DAP or lys to PG is facilitated by the enzyme UDP-N-acetylmuramoylalanyl-D-glutamate-2,6-diaminopimelate ligase (MurE) (EC 6.3.2.13) (Figure 1). Given that the third amino acid is involved in cross-linking, the lack of or incorrect incorporation of either *m*-DAP or lys would lead to improper construction of the cell wall and the inability of the bacterium to withstand the internal osmotic pressure would ultimately lead to cell death via cell lysis.

Inhibition of enzymes involved in the DAP/lys bacterial pathways would have a detrimental effect from two perspectives. Firstly, since DAP or lys is involved in PG cross-linking, cell death via lysis would occur as a result of osmotic pressure from the lack of or improperly constructed PG (Cox, 1996; Baizman et al., 2000). It should be noted that functional PG was recently discovered in chlamydia (Pilhofer et al., 2013). This discovery is very intriguing because existence of PG in chlamydia has been controversial and was known as the chlamydial anomaly due to the fact that Chlamydia was sensitive to antibiotics that were known to inhibit PG synthesis, such as D-cycloserine, bacitracin, and penicillin, yet scientists were not able to detect PG from bacteria belonging to the genus. Secondly, protein synthesis would be suboptimal in a low lys environment, since lys is one of the 20 common amino acid ubiquitously involved in protein synthesis facilitated by lysine-tRNA synthetase (LysU EC 6.1.1.6). It should be noted that compounds that are specific for enzymes involved in the synthesis of DAP/lys pathway would be advantageous, since these enzymes are absent in humans and therefore likely to be non-toxic.

The DapL pathway is of interests given the fact the pathway is narrowly distributed in the bacterial domain based genomic data mining. As such, this pathway provides an opportunity to explore the possibility of inhibiting DapL in organisms that employ this pathway. The DapL pathway is present in *Leptospira interrogans* which colonizes the kidney of mammals and is the culprit of the bacterial disease leptospirosis (Hudson et al., 2008; Evangelista and Coburn, 2010). The DapL pathway has also been identified in the intracellular bacterium *Chlamydia trachomatis* which is responsible for the sexually transmitted disease (STD) “chlamydia” and is considered the most common STD with approximately 90 million new cases of infections yearly (Brunham and Rey-Ladino, 2005; McCoy et al., 2006). In addition, *C. trachomatis* is a major cause of blindness in developing countries (Ghuysen and Goffin, 1999). *Chlamydia pneumonia* is the causative agent in approximately 10% of pneumonia cases in infants (Ghuysen and Goffin, 1999). In this study, the DapL pathway was identified in some species belonging to the genus *Treponema*, which is the causative agent of the STD syphilis (Burstain et al., 1991).

Given the narrow distribution of the DapL pathway in approximately 13% of the genomes of bacteria that have been sequenced and annotated thus far, and the fact that the majority of bacteria employ the *E. coli*-like acyl pathways, DapL is a putative target for narrow spectrum antibacterial compounds. Access to novel narrow spectrum antibacterial compounds that are specific for a certain bacterial species have the potential to avoid risks related to altering the normal microbiome by killing of normal indigenous flora that are involved in the production of vitamins, reabsorption of water and those that are vital for normal physiology among other roles. This is in contrast to broad-spectrum compounds that kill both the pathogen and the beneficial bacteria that are a part of the normal flora (van Saene et al., 1998).

Here we provide a review of the literature pertaining to the discovery of the DapL pathway, structural analyses, inhibitor studies, evolutionary aspects, and the enzyme as a feasible and plausible target for the development of narrow-spectrum antibacterial compounds.

## The discovery of the L,L-diaminopimelate aminotransferase (DapL) variant pathway

The sequence and annotation of the model plant *Arabidopsis thaliana* genome provided the opportunity to do comparative genomics in plants since it was first plant genome to be sequenced and annotated (The Arabidopsis Genome Initiative, 2000). A novel variant of DAP/lys pathway was initially suspected in plants due to the fact that orthologs of dapD, dapC, dapE, and ddh could not be identified in the genome of the plant using bacterial or archael protein sequences as queries against the Arabidopsis genome using tblastn, which is an algorithm that searches translated nucleotides using protein sequences. However, suitable orthologs for the other genes known to be involved in the DAP/lys (dapA, dapB, dapF, and lysA) pathway were readily identified (Hudson et al., 2005). In addition, enzymatic activity of DapD and DapC could not be detected from crude protein extracts prepared from *Glycine max*, *Zea mays*, *Nictotiana tobacum*, and *Chlamydomonas reinhardtii*, yet the extract from *Z. mays* was able to synthesize lys when the precursor dihydrodipicolinate (DHP) was provided as a substrate (Hudson et al., 2005). The analyses from this study suggested that plants bridge the metabolic gap between THDP and L,L-DAP using a putative aminotransferase enzyme. The aminotransferase responsible for this activity was subsequently identified and characterized from *A. thaliana* (locus tag At4g33680) and the cyanobacterium *Syncechocystis* sp. PCC 6803 (locus tag sll0480) using *in vitro* and *in vivo* analyses (Hudson et al., 2006). The fact the gene was identified in a cyanobacterial species meant that this pathway was a feature of photosynthetic cohorts in addition to lineages belonging to the bacterial domain.

## Kinetic analysis of DapL enzymes

Aminotransferases transfer an amino group plus a proton and an electron pair from a donor molecule, usually an amino acid, to the carbonyl position of an acceptor molecule, usually a keto acid (Braunstein, 1973). Kinetic studies of aminotransferases demonstrated that kinetic mechanism is a two-step process following the bimolecular ping-pong model (Velick and Vavra, 1962). Chemically, in the first step the amino donor forms a Schiff based structure to pyridoxal-5′-phosphate (PLP). PLP is covalently linked to a conserved lysine residue in the active site of the enzyme and is a general feature of most aminotransferases (Alexanxer et al., 1994). In the second step, the 2-keto acid acceptor is converted to its amino acid cognate when the amino group from the donor is transferred to the exposed keto group on the acceptor (Leipman and Olsen, 2004).

The kinetic properties of several DapL orthologs have been elucidated to date employing a coupled assay system (Hudson et al., 2006). In the forward assay, glutamate serves as the amino donor and THDP serves as the amino acceptor. In the reverse assay, L,L-DAP serves as the amino donor and 2-oxoglutarate (2OG) serves as the amino acceptor (Hudson et al., 2006). The kinetic properties of eight DapL orthologs with respect to the V_max_ of the forward and reverse directions and the K_m_ for the four substrates are present in Table 2.

**Table 2:** **Kinetic properties of DapL orthologs**.

**Enzyme**	**V_max_ (Forward)**	**V_max_(Reverse)**	**K_m_(L,L-DAP) μM**	**K_m_ (THDP) μM**	**K_m_ (2-OG) mM**	**K_m_ (Glu) mM**	**Citations**
*At*DapL	0.38	22.3	67.0	38.0	8.7	1.9	Hudson et al., 2006
*Ct*DapL	0.01	0.58	116	19.0	2.1	4.0	McCoy et al., 2006
*Pc*DapL	0.09	1.84	6.0	5.0	1.1	0.4	McCoy et al., 2006
*Cr*DapL	0.68	11.6	300.0	100.0	2.2	0.9	Dobson et al., 2011
*Li*DapL	0.45	10.6	37.0	14.0	0.4	4.3	Hudson et al., 2008
*Mt*DapL	0.10	6.30	82.0	7.8	2.6	1.1	Hudson et al., 2008
*Dh*DapL	0.007	0.4	38.2	9.1	0.7	10.1	Hudson et al., 2008
*Mt*DapL^*^	0.006	0.25	60.4	14.0	0.3	4.2	Hudson et al., 2008

The V_max_ for the forward and reverse are in μmol min^−1^ mg^−1^ of purified recombinant protein. The enzyme abbreviations are as follows AtDapL (Arabidopsis thaliana), CtDapL (Chlamydia trachomatis), PcDapL (Protochlamydia amoebophila), CrDapL (Chlamydomonas reinhardtii), LiDapL (Leptospira interrogans), MtDapL (Methanothermobacter thermautotrophicus), DhDapL (Desulfitobacterium hafniense), and MtDapL^*^ (Moorella thermoacetica).

One feature that is apparent from the kinetic analyses is that the enzyme activity with respect to the V_max_ is more robust in the catabolic (reverse) direction when compared to the anabolic (forward) direction. The enzyme is approximately 58 and 24 times more efficient for the *C. trachomatis* and *L. interrogans* orthologs respectively (Table 2). This observation is a general feature of all DapL orthologs that have been fully characterized to date. Initial *in vivo* analysis of the plant ortholog showed that the enzyme is capable of bridging the metabolic gap between THDP and L,L-DAP using functional complementation of the *dap* auxotrophic mutants, namely *dapD*, *dapE*, and *dapD/E* despite being approximately 59 times more efficient in the reverse direction. These assays showed that DapL, when expressed in *E. coli* cells harboring deletion mutations in the *dapD* and *dapE* genes, complemented these mutations when cultured on DAP free media, since the pathway was to facilitate PG and protein synthesis (Hudson et al., 2006).

## Structural analysis of DapL orthologs

**Figure 3:**
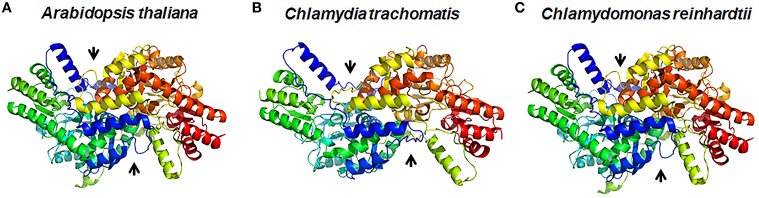
**Three -dimensional representation of DapL orthologs showing the dimeric structure of the enzyme from **(A)** the plant *Arabidopsis thaliana* (PDB id: 3E17), **(B)** the bacterium *Chlamydia trachomatis* (PDB id: 3ASA), and **(C)** the alga *Chlamydomonas reinhardtii* (PDB id: 3QGU)**. The arrows show the two active sites in the dimer (Adapted from McKinnie et al., 2014).

DapLs are approximately 400 amino acids in length and belong to the pyridoxal-5′-phosphate (PLP) dependent family of class I/II aminotransferases. It was demonstrated through phylogenetic analysis that there are two divergent forms of the enzyme namely the Type I and Type II. The two types share approximately 30% homology on the amino acid level (Hudson et al., 2008). The structures of three DapLs have been solved to date using x-ray crystallography. The *A. thaliana* ortholog was the first DapL ortholog to be structurally characterized (Watanabe et al., 2007) followed by the structure of the ortholog from the alga *C. reinhardtii* and subsequently from *C. trachomatis* (Dobson et al., 2011; Watanabe et al., 2011). The overall 3-dimenisional structures of the DapL orthologs that have been solved are depicted in Figures 3A–C. The crystal structures revealed that the holoenzyme is active as a dimer, which is consistent with the majority of aminotransferases (Watanabe et al., 2007). The plant enzyme also revealed that each monomeric unit consisted of two domains namely large domain (LD) and small domain (SD) both of which belong to a α/β class (Watanabe et al., 2007). The α/β structure of each monomer represents a V-shaped conformation that comprises the PLP fold involved in the transamination process. A malate-bound structure of the Arabidopsis ortholog facilitated the modeling of two substrates L,L-DAP and glutamate into the active site of the enzyme was instrumental in elucidating amino acid that are involved in the active site of the enzyme (Watanabe et al., 2007). Interestingly, unlike the Arabidopsis and Chlamydomonas ortholog, the Chlamydia ortholog was found to be promiscuous regarding substrate specificity due to the fact that the enzyme was able to use both racemic DAP isomers as substrates (McCoy et al., 2006). The crystal structure of the DapL ortholog from *C. trachomatis* revealed that this promiscuity could be the result of active-site flexibility due to the open conformation having a 9.5 Å degree of opening of the SD of the monomer making a wider V-shaped active site (Figure 3B). This open SD conformation allows the hinge region more flexibility, which could lead to substrate specificity when compared to the *A. thaliana* and *C. reinhardtii* orthologs (Dobson et al., 2011; Watanabe et al., 2011).

## Inhibitor studies of DapL

Given the fact that DapL is a plausible target for the development of antibacterial compounds, herbicides or algaecides, it was logical that inhibitor strategies would be explored. To that end, 29,201 drug-like compounds were screened against the Arabidopsis ortholog (Fan et al., 2010). The IC_50_ values of 46 of the compounds were determined based on the fact that they were able to inhibit enzyme activity of at least 13% (Fan et al., 2010). In addition, aryl hydrazide and rhodanine modifications were used for the generation of 20 additional analogs in an attempt to elucidate structure-activity relationship (SAR), which are useful in guiding the development of drugs that could be potential biocides (Fan et al., 2010). One of the compounds identified in the initial screen was an *o*-sulfonamido-arylhydrazide, which is a reversible inhibitor with an IC_50_ ~ 5 μM. Further modifications of *o*-sulfonamido-arylhydrazide led to the identifications of compounds that are have increased inhibitory characteristics (Fan and Vederas, 2012). Structural modeling and protein alignment of DapLs from *V. spinosum*, *L. interrogans* using the structural data from the *A. thaliana*, *C. trachomatis*, and *C. reinhardtii* orthologs showed that the amino acids that comprise the active site of DapL are conserved. In addition, this analysis revealed that the overall active site shape was very similar for the five orthologs. Despite this similarity on the structural level, it was shown that there is differential response of DapL to antibiotic lead compounds based on the IC_50_ values ranging from 4.7 to 250 μM the orthologs using inhibitors with structural moiety of a hydrazide, rhodanine, barbiturate and thiobarbiturate (McKinnie et al., 2014). This could perhaps be explained by the protein dynamics; the orthologs share an average structure, as determined by x-ray crystallography, but deviate in the proportion of substructures. It should be noted that it is still unclear whether the inhibitors of DapL that have been discovered thus far are specific for DapL or inhibit aminotransferases in general. In addition, the inhibitor/enzyme interactions pertaining to the actual binding site(s) of the inhibitor to the enzyme are still not clear.

## The distribution of DapL in the domain bacteria

**Figure 4:**
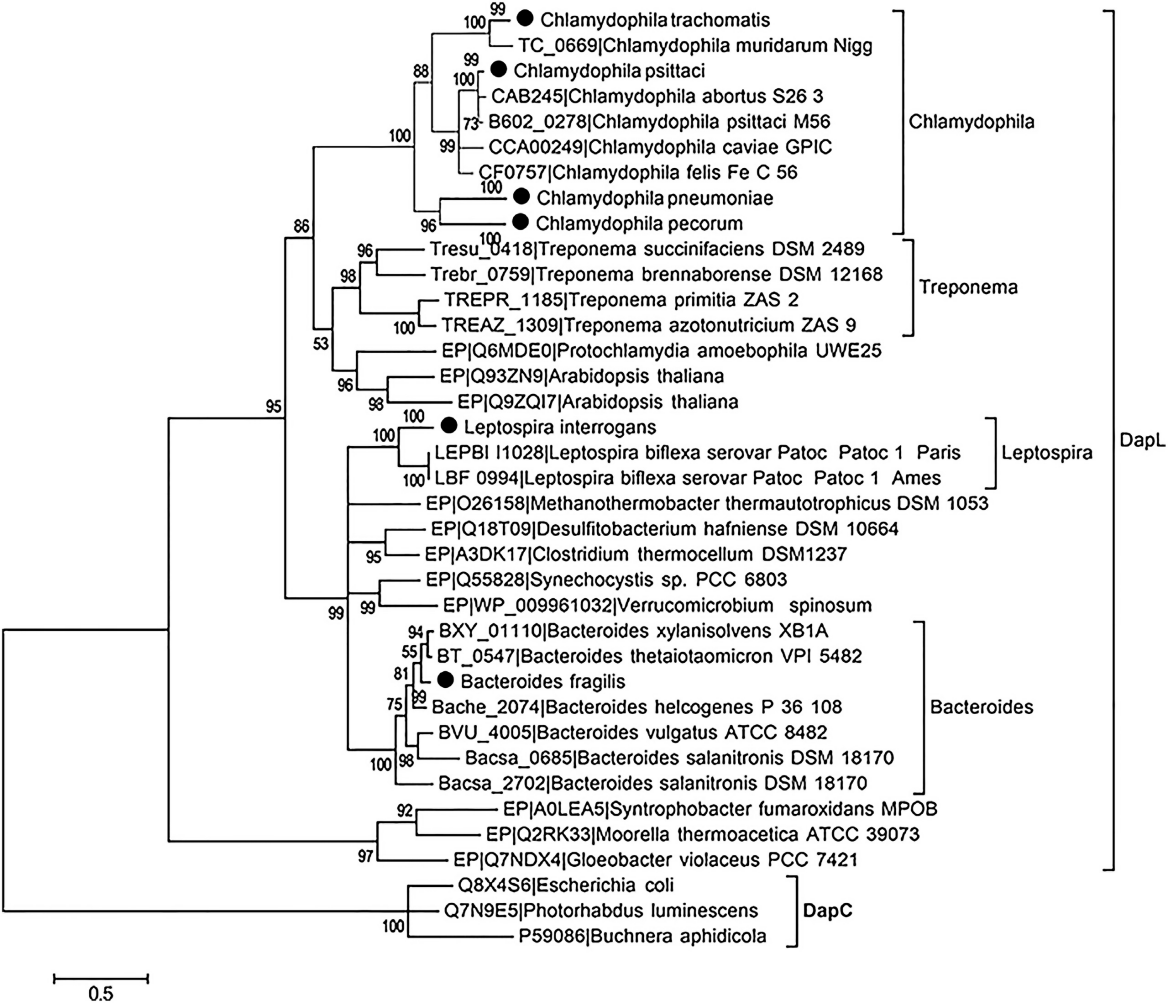
**The evolutionary history of selected DapL as inferred by using the Maximum Likelihood method based on the LG model**. Branches corresponding to partitions that were reproduced in less than 50% of aLRT SH-like supports are collapsed. Clades containing more than 4 taxa with similar species name are compressed and indicated with a black circle next to the node. The tree is drawn to scale, with branch lengths measured in the number of substitutions per site. DapC sequences were chosen as out group and used to root the tree. DapL with the prefix “EP” have been experimentally validated in previous studies (Hudson et al., 2006, 2008, 2011; McCoy et al., 2006; Nachar et al., 2012).

**Figure 5:**
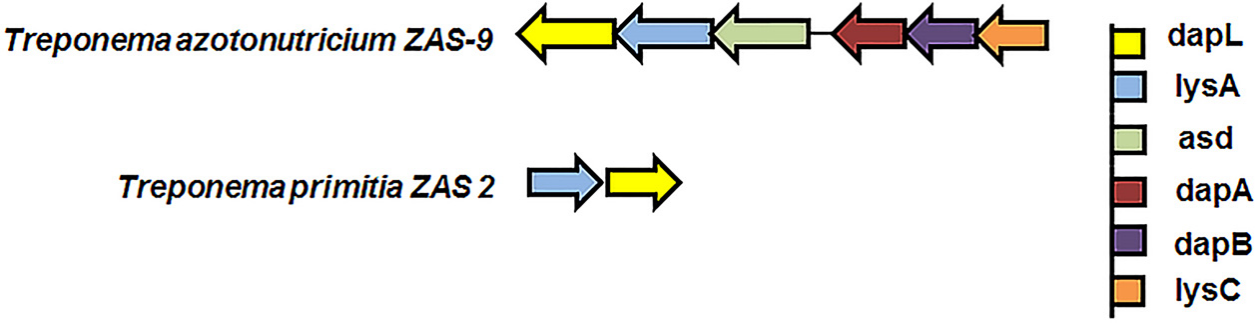
**Genomic context of DAP/lys genes from the bacteria *Treponema azotonutricium* ZAS-9 and *Treponema primitia* ZAS 2**. The information was obtained by searching the Integrated Microbial Genome database (https://img.jgi.doe.gov/cgi-bin/w/main.cgi) using the locus tags TREAZ_1309 and TREPR_1185. The color of each arrow represents a particular gene as denoted in the figure.

To assess the DapL frequency in the bacterial domain, bacterial proteins were extracted and downloaded from the microbial complete genome from the NCBI database as of 5/23/2014. Using the Interproscan5 (Jones et al., 2014), the proteins were then scanned for domains and the resulting list was queried for hits containing IPR019942 which is a signature protein domain for DapL proteins. This search resulted in the identification of 381 out of 2771 bacteria that possessed at least one putative DapL (Supplementary Table 1). The analysis expanded the list of bacterial lineages that were known to contain the DapL pathway from only a few years ago when the pathway was only found in lineages of *Cyanobacteria*, *Desulfuromonadales*, *Firmicutes*, *Bacteriodes*, *Chalmydiae*, *Spirochaeta*, and *Chloroflexi* (Hudson et al., 2008; Fan and Vederas, 2012). The phylogenetic relationship of DapL orthologs associated with pathogens and those with evidence at the protein levels are depicted in Figure 4. Interestingly, the phylogenic tree includes four species from the genus *Treponema*, which are known to be involved in human pathogenesis (Burstain et al., 1991). DapL has not been experimentally confirmed in any species belonging to *Treponema*. Given that it is an important bacterial lineage regarding pathogenicity, a genome context scan was performed to assess if the dapL gene identified in the species were in an operon like structure with genes that are known to be involved in DAP/lys anabolism. The result from this analysis show that the putative dapL orthologs from *Treponema azotonutricum* and *Treponema primitia* are proximal to gene(s) that are known to be involved in DAP/lys synthesis. The genomic context in *Treponema azotonutricum* is very convincing and suggests that the identified ortholog is probably an authentic dapL given the fact that the ortholog is proximal to five other genes that are involved in DAP/lys synthesis missing only the dapF in the structure to complete the synthesis of DAP/lys. It should be noted that the dapF gene is present in another location in the genome of *T. azotonutricum* to complete the synthesis of DAP/lys *de novo* (Figure 5). The DapL orthologs from *T. azotonutricum* and *T. primitia* share 54 and 52% identical to the *A. thaliana* ortholog respectively.

## DapL as a target for narrow-spectrum antibacterial development

There are several factors that make DapL a feasible and plausible target for antibiotic development. Firstly, the enzyme is involved in a pathway that synthesizes an essential amino acid and PG, which are both absent in the humans. In addition, the pathway is narrowly distributed, which makes it a putative target for a narrow-spectrum antibiotic. This is because the majority of bacteria utilize the acyl pathways for DAP/lys synthesis and inhibition of the other enzymes in the pathway have the potential to be very detrimental regarding the killing of beneficial bacteria. In addition, in the majority of bacteria that contain dapL, the DapL pathway is the sole route toward DAP/lys synthesis (Hudson et al., 2008; Nachar et al., 2012). The only exceptions that have been experimentally confirmed are in the genomes of *Bacterioides fragilis* and *Clostridium thermocellum*, which were found to contain both the Ddh and DapL pathways (Hudson et al., 2011). A search of the bacterial proteomes using the protein signature IPR010190, which is a signature of Ddh, revealed that *C. trachomatis*, *L. interrogans*, and *Treponema* sp. *do not* contain a Ddh enzyme in addition to the acyl pathway enzymes. It should be noted that some aminotransferases are promiscuous regarding substrate specificity. For example, the *E. coli* aspartate aminotransferase, tyrosine aminotransferase, and the branched-chain aminotransferase have been shown to have overlapping activities (Gu et al., 1998). In addition, a recent study demonstrated that three aminotransferases are involved in alanine biosynthesis in *E. coli* (Yoneyama et al., 2011). Therefore, whether other aminotransferases exist in eubacteria that are capable of catalyzing the direct conversion of THDP to L,L-DAP to facilitate PG and lys biosynthesis is still not known and awaits experimentation. If the dapL gene is essential in eubacteria as it is in plants then the enzyme would be plausible target for antibacterial development.

**Figure 6:**
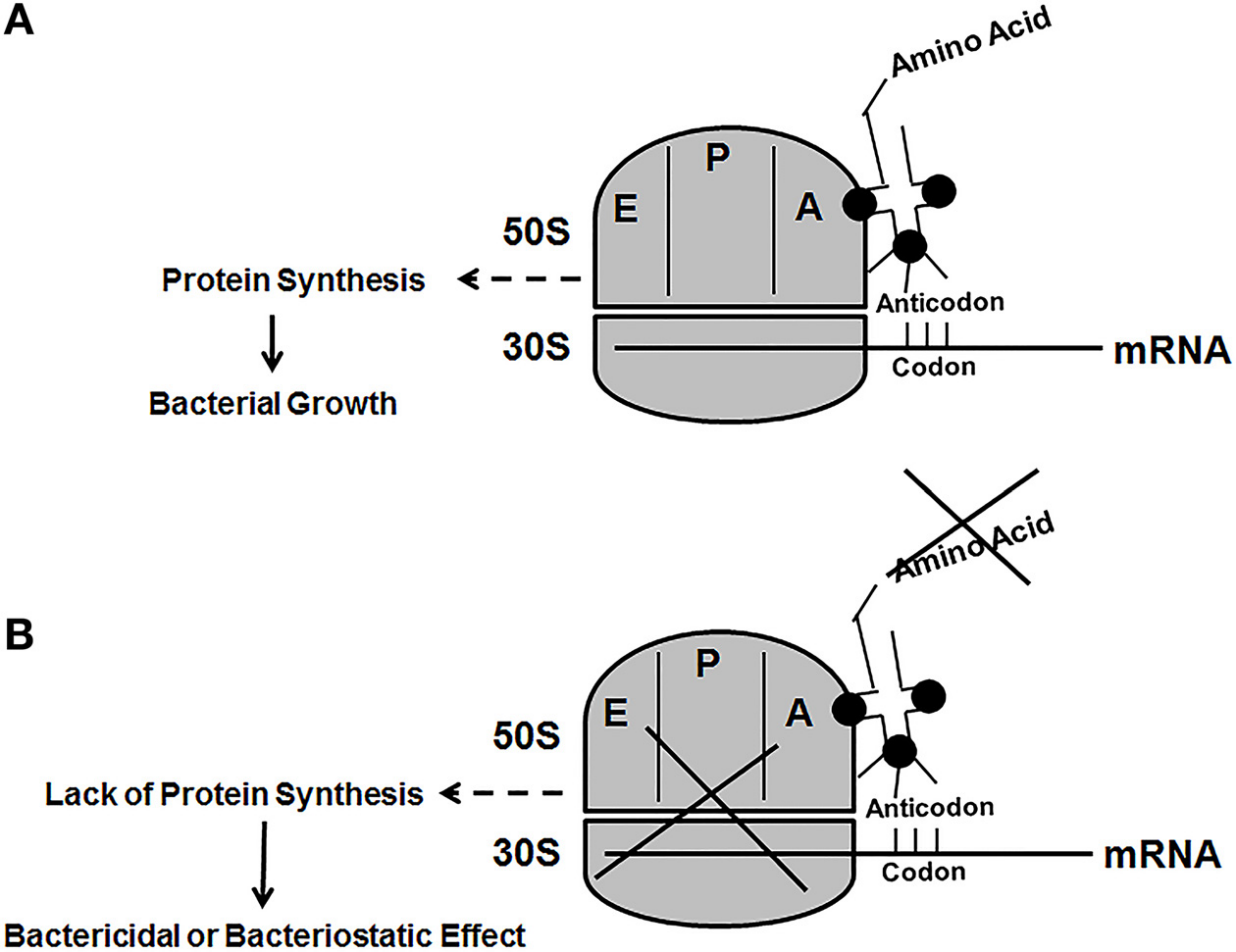
**The figure is a schematic representation of the protein translational system in bacteria. (A)** The normal process where the tRNA charged with the appropriate amino acid (the fuel) is able to facilitate protein synthesis (the engine). **(B)** Bactericidal or bacteriostatic effect due to lack of the appropriate amino acid to facilitate protein synthesis. The 50S and 30S ribosomal subunits are shown with the E (exit site), P (peptidyl site), and the A (aminoacyl site).

A point that should be taken into consideration is the possibility of bacteria acquiring lys from the host using amino acid transport systems, which are ubiquitously found in prokaryotes (Burkovski and Krämer, 2002). A lys specific transporter encoded by the lysP gene exists in *E. coli*. This permease belongs to the amino acid, polyamine, and organocation (APC) transporter family (Steffes et al., 1992). Although it is possible to synthesize lys from DAP, it is not possible to synthesize DAP from lys to facilitate PG biosynthesis in bacteria that require *m*-DAP as the cross-linking amino acid. This would require an enzyme (lysine carboxylase) capable of adding a carboxyl group to lys to make *m*-DAP, which to our knowledge has never been described. In addition, the inhibition of lys biosynthesis would preclude the bacteria from synthesizing permeases since lys is one of the amino acids that is needed to construct these proteins. For example, the LysP protein from *E. coli K-12* substs MG1655 (NP_416661) is 489 amino acids and contains 14 lys residues. Another point that should be considered is that even if the bacterium is able to sequester lys through passive transport means, the rate of incorporation would unlikely to be equal to the rate of protein synthesis. This scenario would probably lead to stalling of the translational system due to the fact that lys would not be readily available to charge the lys specific tRNA to facilitate protein synthesis. Under this condition it would lead to either a bactericidal or bacteriostatic effect (Figure 6).

## Summary

The protein translation machinery is an established target for antibacterial compounds given the fact that antibiotic classes, such as tetracyclines, aminoglycodies, streptogramins, macrolides, and lincosamides, inhibit the ribosomal machinery which we define as “the engine” of the translation system by specifically binding to the 30S ribosomal subunit (Davies and Davies, 2010) (Table 3). This raises the question of whether it is plausible and feasible to inhibit the incorporation of amino acids which we define in this context as “the fuel” for the system specifically the essential amino acids as a mean of antibacterial development. An essential amino acid such as lysine would be a novel antibacterial target since the current targets are relegated to PG biosynthesis, translation, DNA replication, C1 metabolism, transcription, and cell membrane (Table 3). The idea of amino acid biosynthesis inhibition as a biocide is not novel. In fact, the inhibition mechanism of the herbicide glyphosate commonly referred to as Roundup™ was elucidated in the early 1970s by Monsanto scientists and was shown to be a nonselective herbicide that inhibits the enzyme enolpyruvylshikimate phosphate synthase which is involved in the synthesis of the amino acids, tyrosine (tyr), phenylalanine (phe), and tryptophan (trp) (Williams et al., 2000). It should be noted that the pathway for tyr, phe, and trp through to the central metabolite chorismate is not present in the animal kingdom which makes the glyphosate a superior herbicide and being non-toxic to humans. As such, the lack of genetic machineries to anabolize the essential amino acids provide an opportunity to explore the feasibility and plausibility of identifying and or developing compounds that can be used as chemotherapies as antibacterial agents. These compounds would be advantageous in combating the rise in antibiotic resistant bacteria in addition to emerging infections and diseases.

**Table 3:** **Families, targets, and examples of antibiotics (Adapted from Davies and Davies, 2010)**.

**Families**	**Target**	**Example(s)**
β-lactams	Peptidoglycan biosynthesis	Penicillins
Glycopeptides		Cephalosporins
		Penems
		Monobactams
		Vancomycin
		Teicoplanin
Tetracyclines	Translation	Minocycline
Aminoglycosides		Tigecycline
Streptogramins		Gentamicin
Macrolides		Streptomycin
Lincosamides		Spectinomycin
		Synercid
		Erythromycin
		Azithromycin
		Clindamycin
Quinolones	DNA replication	Ciprofloxacin
Pyrimidines	C_1_ metabolism	Trimethoprim
Sulfonamides		Sulfamethoxazole
Rifamycins	Transcription	Rifampin
Lipopeptides	Bacterial cell membrane	Daptomycin
Cationic peptides		Colistin

### Conflict of interest statement

The authors declare that the research was conducted in the absence of any commercial or financial relationships that could be construed as a potential conflict of interest.
