# Supplementary material for: L,L-diaminopimelate aminotransferase (DapL): a putative target for the development of narrow-spectrum antibacterial compounds
Source: Front Microbiol. 2014 Sep 26;5:509. doi: 10.3389/fmicb.2014.00509 (PMC4176475; doi:10.3389/fmicb.2014.00509)
Supplement: Supplementary file 1 [file Table1.DOCX]

**Supplementary Table 1.** Putative DapL identified by performing a complete interproscan5 analysis on the protein sequences from the complete microbial genomes in NCBI (<ftp://ftp.ncbi.nlm.nih.gov/genomes/Bacteria/>) as of 5/23/2014 and subsequently filtering the result for proteins associated with the interproscan ID IPR019942 which corresponds to the signatures from PANTHER database [PTHR11751:SF22], HAMAP database [MF_01642 (DapL_aminotrans_1)] and TIGRFAMS database [TIGR03542 (DAPAT_plant)].

| **Organism** | **Locus Tag** | **Protein Annotation Description** |
| --- | --- | --- |
| *Acaryochloris marina MBIC11017 uid58167* | AM1_1880 | L,L-diaminopimelate aminotransferase |
| *Acetobacterium woodii DSM 1030 uid88073* | Awo_c17620 | Aspartate aminotransferase AspB |
| *Acidaminococcus fermentans DSM 20731 uid43471* | Acfer_0333 | L,L-diaminopimelate aminotransferase |
| *Acidaminococcus intestini RyC MR95 uid74445* | Acin_0020 | L,L-diaminopimelate aminotransferase |
| *Adlercreutzia equolifaciens DSM 19450 uid223286* | AEQU_0414 | L,L-diaminopimelate aminotransferase |
| *Akkermansia muciniphila ATCC BAA 835 uid58985* | Amuc_1185 | L,L-diaminopimelate aminotransferase |
| *Anabaena 90 uid179383* | ANA_C11356 | L,L-diaminopimelate aminotransferase |
| *Anabaena cylindrica PCC 7122 uid183339* | Anacy_4955 | L,L-diaminopimelate aminotransferase apoenzyme |
| *Anabaena variabilis ATCC 29413 uid58043* | Ava_1277 | L,L-diaminopimelate aminotransferase |
| *Anabaena variabilis ATCC 29413 uid58043* | Ava_2354 | L,L-diaminopimelate aminotransferase |
| *Aquifex aeolicus VF5 uid57765* | aq_273 | L,L-diaminopimelate aminotransferase |
| *Arthrospira platensis NIES 39 uid197171* | NIES39_E01700 | aminotransferase |
| *Arthrospira platensis NIES 39 uid197171* | NIES39_J05350 | aminotransferase |
| *Bacteroides fragilis 638R uid84217* | BF638R_2671 | Putative aminotransferase-related protein |
| *Bacteroides fragilis NCTC 9343 uid57639* | BF9343_2584 | Putative aminotransferase-related protein |
| *Bacteroides fragilis YCH46 uid58195* | BF2643 | Aspartate aminotransferase |
| *Bacteroides helcogenes P 36 108 uid62135* | Bache_2074 | L,L-diaminopimelate aminotransferase apoenzyme |
| *Bacteroides salanitronis DSM 18170 uid63269* | Bacsa_0685 | L,L-diaminopimelate aminotransferase |
| *Bacteroides salanitronis DSM 18170 uid63269* | Bacsa_2702 | L,L-diaminopimelate aminotransferase |
| *Bacteroides thetaiotaomicron VPI 5482 uid62913* | BT_0547 | L,L-diaminopimelate aminotransferase |
| *Bacteroides vulgatus ATCC 8482 uid58253* | BVU_4005 | L,L-diaminopimelate aminotransferase |
| *Bacteroides xylanisolvens XB1A uid197168* | BXY_01110 | L,L-diaminopimelate aminotransferase apoenzyme |
| *butyrate producing bacterium SS3 4 uid197159* | CK3_27440 | L,L-diaminopimelate aminotransferase apoenzyme |
| *butyrate producing bacterium SSC 2 uid197181* | CL2_09750 | L,L-diaminopimelate aminotransferase apoenzyme |
| *Butyrivibrio fibrisolvens uid197155* | CIY_02000 | L,L-diaminopimelate aminotransferase apoenzyme |
| *Butyrivibrio proteoclasticus B316 uid51489* | bpr_I2331 | L,L-diaminopimelate aminotransferase |
| *Calothrix PCC 6303 uid183109* | Cal6303_3493 | L,L-diaminopimelate aminotransferase apoenzyme |
| *Calothrix PCC 7507 uid182930* | Cal7507_1023 | L,L-diaminopimelate aminotransferase apoenzyme |
| *Calothrix PCC 7507 uid182930* | Cal7507_2942 | L,L-diaminopimelate aminotransferase apoenzyme |
| *Candidatus Azobacteroides pseudotrichonymphae genomovar CFP2 uid59163* | CFPG_571 | L,L-diaminopimelate aminotransferase |
| *Desulforudis audaxviator MP104C uid59067* | Daud_1603 | L,L-diaminopimelate aminotransferase |
| *Methylomirabilis oxyfera uid161981* | DAMO_2313 | PLP-dependent aminotransferase, Putative Aspartate aminotransferase |
| *Nitrospira defluvii uid51175* | NIDE0503 | aminotransferase |
| *Protochlamydia amoebophila UWE25 uid58079* | pc0685 | L,L-diaminopimelate aminotransferase |
| *Carboxydothermus hydrogenoformans Z 2901 uid57821* | CHY_1492 | L,L-diaminopimelate aminotransferase |
| *Chamaesiphon PCC 6605 uid183005* | Cha6605_4159 | L,L-diaminopimelate aminotransferase |
| *Chlamydia muridarum Nigg uid57785* | TC_0669 | aminotransferase, class I |
| *Chlamydia pecorum P787 uid221292* | CPE3_0254 | Putative aminotransferase |
| *Chlamydia pecorum PV3056 3 uid221290* | CPE1_0254 | Putative aminotransferase |
| *Chlamydia pecorum W73 uid221291* | CPE2_0254 | Putative aminotransferase |
| *Chlamydia psittaci 01DC12 uid179070* | BN356_2561 | Putative aminotransferase |
| *Chlamydia psittaci 84 55 uid175571* | B595_0285 | L,L-diaminopimelate aminotransferase |
| *Chlamydia psittaci GR9 uid175572* | B598_0279 | L,L-diaminopimelate aminotransferase |
| *Chlamydia psittaci M56 uid175576* | B602_0278 | L,L-diaminopimelate aminotransferase |
| *Chlamydia psittaci MN uid175573* | B599_0277 | L,L-diaminopimelate aminotransferase |
| *Chlamydia psittaci VS225 uid175574* | B600_0292 | L,L-diaminopimelate aminotransferase |
| *Chlamydia psittaci WC uid175577* | B603_0278 | L,L-diaminopimelate aminotransferase |
| *Chlamydia psittaci WS RT E30 uid175575* | B601_0279 | L,L-diaminopimelate aminotransferase |
| *Chlamydia trachomatis 434 Bu uid61633* | CTL0646 | L,L-diaminopimelate aminotransferase |
| *Chlamydia trachomatis A 363 uid196769* | A363_00418 | L,L-diaminopimelate aminotransferase |
| *Chlamydia trachomatis A 5291 uid196770* | A5291_00417 | L,L-diaminopimelate aminotransferase |
| *Chlamydia trachomatis A HAR 13 uid58333* | CTA_0425 | L,L-diaminopimelate aminotransferase |
| *Chlamydia trachomatis A2497 uid159863* | CTR_3891 | L,L-diaminopimelate aminotransferase |
| *Chlamydia trachomatis A2497 uid159993* | CTO_0425 | Aspartate aminotransferase |
| *Chlamydia trachomatis B Jali20 OT uid59351* | JALI_3891 | L,L-diaminopimelate aminotransferase |
| *Chlamydia trachomatis B TZ1A828 OT uid59349* | CTB_3891 | L,L-diaminopimelate aminotransferase |
| *Chlamydia trachomatis C TW 3 uid232251* | CTW3_02130 | Aspartate aminotransferase |
| *Chlamydia trachomatis D EC uid159881* | CTDEC_0390 | Aspartate aminotransferase |
| *Chlamydia trachomatis D LC uid159879* | CTDLC_0390 | Aspartate aminotransferase |
| *Chlamydia trachomatis D SotonD1 uid196772* | SOTOND1_00412 | L,L-diaminopimelate aminotransferase |
| *Chlamydia trachomatis D SotonD5 uid196773* | SOTOND5_00409 | L,L-diaminopimelate aminotransferase |
| *Chlamydia trachomatis D SotonD6 uid196774* | SOTOND6_00409 | L,L-diaminopimelate aminotransferase |
| *Chlamydia trachomatis D UW 3 CX uid57637* | CT_390 | Aspartate Aminotransferase |
| *Chlamydia trachomatis E 11023 uid161369* | E11023_02030 | L,L-diaminopimelate aminotransferase |
| *Chlamydia trachomatis E 150 uid161403* | E150_02045 | L,L-diaminopimelate aminotransferase |
| *Chlamydia trachomatis E Bour uid196775* | BOUR_00415 | L,L-diaminopimelate aminotransferase |
| *Chlamydia trachomatis E SotonE8 uid196777* | SOTONE8_00416 | L,L-diaminopimelate aminotransferase |
| *Chlamydia trachomatis E SW3 uid167483* | ESW3_3961 | L,L-diaminopimelate aminotransferase |
| *Chlamydia trachomatis F SW4 uid167484* | FSW4_3961 | L,L-diaminopimelate aminotransferase |
| *Chlamydia trachomatis F SW5 uid167485* | FSW5_3961 | L,L-diaminopimelate aminotransferase |
| *Chlamydia trachomatis G 11074 uid161409* | G11074_02015 | L,L-diaminopimelate aminotransferase |
| *Chlamydia trachomatis G 11222 uid161361* | G11222_02015 | L,L-diaminopimelate aminotransferase |
| *Chlamydia trachomatis G 9301 uid161377* | CTG9301_02020 | L,L-diaminopimelate aminotransferase |
| *Chlamydia trachomatis G 9768 uid161353* | G9768_02015 | L,L-diaminopimelate aminotransferase |
| *Chlamydia trachomatis G SotonG1 uid196779* | SOTONG1_00410 | L,L-diaminopimelate aminotransferase |
| *Chlamydia trachomatis Ia SotonIa1 uid196780* | SOTONIA1_00411 | L,L-diaminopimelate aminotransferase |
| *Chlamydia trachomatis IU824 uid193712* | BN197_3941 | L,L-diaminopimelate aminotransferase |
| *Chlamydia trachomatis IU888 uid193713* | BN442_3941 | L,L-diaminopimelate aminotransferase |
| *Chlamydia trachomatis J 6276tet1 uid213394* | CTJTET1_02040 | L,L-diaminopimelate aminotransferase |
| *Chlamydia trachomatis K SotonK1 uid196782* | SOTONK1_00409 | L,L-diaminopimelate aminotransferase |
| *Chlamydia trachomatis L1 115 uid196784* | L1115_00405 | L,L-diaminopimelate aminotransferase |
| *Chlamydia trachomatis L1 224 uid196785* | L1224_00405 | L,L-diaminopimelate aminotransferase |
| *Chlamydia trachomatis L1 440 LN uid196783* | L1440_00407 | L,L-diaminopimelate aminotransferase |
| *Chlamydia trachomatis L2 25667R uid196786* | L225667R_00406 | L,L-diaminopimelate aminotransferase |
| *Chlamydia trachomatis L2 434 Bu f uid198644* | CTLFINAL_03375 | L,L-diaminopimelate aminotransferase |
| *Chlamydia trachomatis L2 434 Bu i uid198643* | CTLINITIAL_03365 | L,L-diaminopimelate aminotransferase |
| *Chlamydia trachomatis L2b 795 uid196791* | L2B795_00405 | L,L-diaminopimelate aminotransferase |
| *Chlamydia trachomatis L2b 8200 07 uid196787* | L2B8200_00404 | L,L-diaminopimelate aminotransferase |
| *Chlamydia trachomatis L2b Ams1 uid196792* | L2BAMS1_00404 | L,L-diaminopimelate aminotransferase |
| *Chlamydia trachomatis L2b Ams2 uid196793* | L2BAMS2_00404 | L,L-diaminopimelate aminotransferase |
| *Chlamydia trachomatis L2b Ams3 uid196794* | L2BAMS3_00404 | L,L-diaminopimelate aminotransferase |
| *Chlamydia trachomatis L2b Ams4 uid196795* | L2BAMS4_00405 | L,L-diaminopimelate aminotransferase |
| *Chlamydia trachomatis L2b Ams5 uid196796* | L2BAMS5_00405 | L,L-diaminopimelate aminotransferase |
| *Chlamydia trachomatis L2b Canada1 uid196798* | L2BCAN1_00406 | L,L-diaminopimelate aminotransferase |
| *Chlamydia trachomatis L2b Canada2 uid196799* | L2BCAN2_00405 | L,L-diaminopimelate aminotransferase |
| *Chlamydia trachomatis L2b CV204 uid196790* | L2BCV204_00404 | L,L-diaminopimelate aminotransferase |
| *Chlamydia trachomatis L2b UCH 1 proctitis uid61635* | CTLon_0643 | L,L-diaminopimelate aminotransferase |
| *Chlamydia trachomatis L2b UCH 2 uid196788* | L2BUCH2_00404 | L,L-diaminopimelate aminotransferase |
| *Chlamydia trachomatis L2c uid68843* | CTL2C_909 | L,L-diaminopimelate aminotransferase |
| *Chlamydia trachomatis L3 404 LN uid196797* | L3404_00405 | L,L-diaminopimelate aminotransferase |
| *Chlamydia trachomatis RC F 69 uid213385* | CTRC69_02055 | L,L-diaminopimelate aminotransferase |
| *Chlamydia trachomatis RC F s 342 uid213391* | CTRC342_02065 | L,L-diaminopimelate aminotransferase |
| *Chlamydia trachomatis RC F s 852 uid213387* | CTRC852_02080 | L,L-diaminopimelate aminotransferase |
| *Chlamydia trachomatis RC J 943 uid213388* | CTRC943_02025 | L,L-diaminopimelate aminotransferase |
| *Chlamydia trachomatis RC J 953 uid213389* | CTRC953_02025 | L,L-diaminopimelate aminotransferase |
| *Chlamydia trachomatis RC J 966 uid213393* | CTRC966_02035 | L,L-diaminopimelate aminotransferase |
| *Chlamydia trachomatis RC J 971 uid213395* | CTRC971_02025 | L,L-diaminopimelate aminotransferase |
| *Chlamydia trachomatis RC J s 122 uid213392* | CTRC122_02060 | L,L-diaminopimelate aminotransferase |
| *Chlamydia trachomatis RC L2 55 uid213396* | CTRC55_02040 | L,L-diaminopimelate aminotransferase |
| *Chlamydia trachomatis RC L2 s 3 uid213390* | CTRC3_02060 | L,L-diaminopimelate aminotransferase |
| *Chlamydia trachomatis RC L2 s 46 uid213386* | CTRC46_02040 | L,L-diaminopimelate aminotransferase |
| *Chlamydia trachomatis Sweden2 uid161995* | SW2_3961 | L,L-diaminopimelate aminotransferase |
| *Chlamydia trachomatis uid196771* | A7249_00417 | L,L-diaminopimelate aminotransferase |
| *Chlamydia trachomatis uid196776* | SOTONE4_00410 | L,L-diaminopimelate aminotransferase |
| *Chlamydia trachomatis uid196778* | SOTONF3_00410 | L,L-diaminopimelate aminotransferase |
| *Chlamydia trachomatis uid196781* | SOTONIA3_00411 | L,L-diaminopimelate aminotransferase |
| *Chlamydia trachomatis uid196789* | L2BLST_00404 | L,L-diaminopimelate aminotransferase |
| *Chlamydia trachomatis uid196800* | L11322_00405 | L,L-diaminopimelate aminotransferase |
| *Chlamydia trachomatis uid216090* | O169_02140 | Aspartate aminotransferase |
| *Chlamydia trachomatis uid216090* | O170_02130 | Aspartate aminotransferase |
| *Chlamydia trachomatis uid216090* | O172_02125 | Aspartate aminotransferase |
| *Chlamydia trachomatis uid216090* | O173_02135 | Aspartate aminotransferase |
| *Chlamydia trachomatis uid216090* | O175_02130 | Aspartate aminotransferase |
| *Chlamydia trachomatis uid216090* | O176_02130 | Aspartate aminotransferase |
| *Chlamydia trachomatis uid216090* | O177_02120 | Aspartate aminotransferase |
| *Chlamydia trachomatis uid216090* | O178_02125 | Aspartate aminotransferase |
| *Chlamydia trachomatis uid216090* | O179_02125 | Aspartate aminotransferase |
| *Chlamydia trachomatis uid216090* | O180_02125 | Aspartate aminotransferase |
| *Chlamydophila abortus S26 3 uid57963* | CAB245 | L,L-diaminopimelate aminotransferase |
| *Chlamydophila caviae GPIC uid57783* | CCA00249 | L,L-diaminopimelate aminotransferase |
| *Chlamydophila felis Fe C 56 uid57971* | CF0757 | L,L-diaminopimelate aminotransferase |
| *Chlamydophila pecorum E58 uid66295* | G5S_0579 | Aspartate transaminase |
| *Chlamydophila pneumoniae CWL029 uid57811* | CPn0495 | L,L-diaminopimelate aminotransferase |
| *Chlamydophila pneumoniae J138 uid57829* | CPj0495 | L,L-diaminopimelate aminotransferase |
| *Chlamydophila pneumoniae LPCoLN uid159529* | CPK_ORF01011 | class I and II aminotransferase |
| *Chlamydophila pneumoniae TW 183 uid57997* | CpB0515 | L,L-diaminopimelate aminotransferase |
| *Chlamydophila psittaci 01DC11 uid159527* | CPS0A_0281 | L,L-diaminopimelate aminotransferase |
| *Chlamydophila psittaci 02DC15 uid159521* | CPS0B_0277 | L,L-diaminopimelate aminotransferase |
| *Chlamydophila psittaci 08DC60 uid159525* | CPS0D_0279 | L,L-diaminopimelate aminotransferase |
| *Chlamydophila psittaci 6BC uid159845* | G5O_0281 | class I aminotransferase |
| *Chlamydophila psittaci 6BC uid63621* | CPSIT_0275 | L,L-diaminopimelate aminotransferase |
| *Chlamydophila psittaci C19 98 uid159523* | CPS0C_0280 | L,L-diaminopimelate aminotransferase |
| *Chlamydophila psittaci CP3 uid175578* | B711_0286 | L,L-diaminopimelate aminotransferase |
| *Chlamydophila psittaci Mat116 uid189026* | AO9_01340 | L,L-diaminopimelate aminotransferase |
| *Chlamydophila psittaci NJ1 uid175579* | B712_0278 | L,L-diaminopimelate aminotransferase |
| *Chlamydophila psittaci RD1 uid162063* | Cpsi_2611 | Putative aminotransferase |
| *Chroococcidiopsis thermalis PCC 7203 uid183002* | Chro_2418 | L,L-diaminopimelate aminotransferase apoenzyme |
| *Clostridium autoethanogenum DSM 10061 uid225029* | CAETHG_3510 | L,L-diaminopimelate aminotransferase |
| *Clostridium BNL1100 uid84307* | Clo1100_0829 | L,L-diaminopimelate aminotransferase |
| *Clostridium cf saccharolyticum K10 uid197201* | CLS_18990 | L,L-diaminopimelate aminotransferase apoenzyme |
| *Clostridium clariflavum DSM 19732 uid82345* | Clocl_3278 | L,L-diaminopimelate aminotransferase apoenzyme |
| *Clostridium lentocellum DSM 5427 uid49117* | Clole_4248 | L,L-diaminopimelate aminotransferase |
| *Clostridium ljungdahlii DSM 13528 uid50583* | CLJU_c14280 | aromatic animotransferase |
| *Clostridium phytofermentans ISDg uid58519* | Cphy_0631 | L,L-diaminopimelate aminotransferase |
| *Clostridium saccharolyticum WM1 uid51419* | Closa_1275 | L,L-diaminopimelate aminotransferase |
| *Clostridium stercorarium DSM 8532 uid186819* | Cst_c09590 | L,L-diaminopimelate aminotransferase DapL |
| *Clostridium stercorarium DSM 8532 uid195569* | Clst_0919 | diaminopimelate aminotransferase |
| *Clostridium SY8519 uid68705* | CXIVA_05550 | PLP-dependent aminotransferase |
| *Clostridium thermocellum ATCC 27405 uid57917* | Cthe_3101 | L,L-diaminopimelate aminotransferase |
| *Clostridium thermocellum DSM 1313 uid161989* | Clo1313_0652 | L,L-diaminopimelate aminotransferase |
| *Coprococcus ART55 1 uid197176* | CCU_03160 | L,L-diaminopimelate aminotransferase apoenzyme |
| *Coprococcus catus GD 7 uid197174* | CC1_06680 | L,L-diaminopimelate aminotransferase apoenzyme |
| *Crinalium epipsammum PCC 9333 uid183113* | Cri9333_0850 | L,L-diaminopimelate aminotransferase apoenzyme |
| *Cyanobacterium PCC 10605 uid183340* | Cyan10605_0344 | L,L-diaminopimelate aminotransferase apoenzyme |
| *Cyanobacterium stanieri PCC 7202 uid183337* | Cyast_2253 | L,L-diaminopimelate aminotransferase apoenzyme |
| *cyanobacterium UCYN A uid43697* | UCYN_09940 | L,L-diaminopimelate aminotransferase apoenzyme |
| *Cyanobium gracile PCC 6307 uid182931* | Cyagr_1874 | L,L-diaminopimelate aminotransferase |
| *Cyanothece ATCC 51142 uid59013* | cce_2605 | L,L-diaminopimelate aminotransferase |
| *Cyanothece PCC 7424 uid59025* | PCC7424_4059 | L,L-diaminopimelate aminotransferase |
| *Cyanothece PCC 7425 uid59435* | Cyan7425_4424 | L,L-diaminopimelate aminotransferase |
| *Cyanothece PCC 7822 uid52547* | Cyan7822_1964 | L,L-diaminopimelate aminotransferase |
| *Cyanothece PCC 8801 uid59027* | PCC8801_0494 | L,L-diaminopimelate aminotransferase |
| *Cyanothece PCC 8802 uid59143* | Cyan8802_0509 | L,L-diaminopimelate aminotransferase |
| *Cylindrospermum stagnale PCC 7417 uid183111* | Cylst_0116 | L,L-diaminopimelate aminotransferase apoenzyme |
| *Cylindrospermum stagnale PCC 7417 uid183111* | Cylst_4990 | Aspartate/tyrosine/aromatic aminotransferase |
| *Dactylococcopsis salina PCC 8305 uid183341* | Dacsa_0864 | L,L-diaminopimelate aminotransferase |
| *Dactylococcopsis salina PCC 8305 uid183341* | Dacsa_2050 | Aspartate/tyrosine/aromatic aminotransferase |
| *Dehalobacter 11DCA uid177715* | DHBDCA_p1440 | L,L-diaminopimelate aminotransferase |
| *Dehalobacter CF uid177714* | DCF50_p1453 | L,L-diaminopimelate aminotransferase |
| *Dehalococcoides BAV1 uid58477* | DehaBAV1_0669 | L,L-diaminopimelate aminotransferase |
| *Dehalococcoides CBDB1 uid58413* | cbdb_A714 | L,L-diaminopimelate aminotransferase |
| *Dehalococcoides ethenogenes 195 uid57763* | DET0739 | L,L-diaminopimelate aminotransferase |
| *Dehalococcoides GT uid42115* | DehalGT_0630 | L,L-diaminopimelate aminotransferase |
| *Dehalococcoides mccartyi BTF08 uid190183* | btf_661 | L,L-diaminopimelate aminotransferase |
| *Dehalococcoides mccartyi DCMB5 uid190184* | dcmb_707 | L,L-diaminopimelate aminotransferase |
| *Deinococcus proteolyticus MRP uid63399* | Deipr_1376 | L,L-diaminopimelate aminotransferase |
| *Desulfarculus baarsii DSM 2075 uid51371* | Deba_1873 | L,L-diaminopimelate aminotransferase |
| *Desulfatibacillum alkenivorans AK 01 uid58913* | Dalk_0414 | class I and II aminotransferase |
| *Desulfitobacterium dehalogenans ATCC 51507 uid82553* | Desde_3918 | L,L-diaminopimelate aminotransferase apoenzyme |
| *Desulfitobacterium dichloroeliminans LMG P 21439 uid82555* | Desdi_3300 | L,L-diaminopimelate aminotransferase apoenzyme |
| *Desulfitobacterium hafniense DCB 2 uid57749* | Dhaf_4564 | L,L-diaminopimelate aminotransferase |
| *Desulfitobacterium hafniense Y51 uid58605* | DSY3402 | L,L-diaminopimelate aminotransferase |
| *Desulfobacca acetoxidans DSM 11109 uid65785* | Desac_2493 | L,L-diaminopimelate aminotransferase |
| *Desulfobacterium autotrophicum HRM2 uid59061* | HRM2_02680 | L,L-diaminopimelate aminotransferase |
| *Desulfobacula toluolica Tol2 uid175777* | TOL2_C03010 | L,L-diaminopimelate aminotransferase (L,L-DAP-aminotransferase) DapL |
| *Desulfobulbus propionicus DSM 2032 uid62265* | Despr_3323 | L,L-diaminopimelate aminotransferase apoenzyme |
| *Desulfocapsa sulfexigens DSM 10523 uid189952* | UWK_03272 | L,L-diaminopimelate aminotransferase apoenzyme |
| *Desulfococcus oleovorans Hxd3 uid58777* | Dole_2779 | L,L-diaminopimelate aminotransferase |
| *Desulfohalobium retbaense DSM 5692 uid59183* | Dret_1351 | L,L-diaminopimelate aminotransferase |
| *Desulfomicrobium baculatum DSM 4028 uid59217* | Dbac_0704 | class I and II aminotransferase |
| *Desulfosporosinus acidiphilus SJ4 uid156759* | Desaci_2203 | L,L-diaminopimelate aminotransferase apoenzyme |
| *Desulfosporosinus meridiei DSM 13257 uid75097* | Desmer_0699 | L,L-diaminopimelate aminotransferase apoenzyme |
| *Desulfosporosinus orientis DSM 765 uid82939* | Desor_0643 | L,L-diaminopimelate aminotransferase |
| *Desulfotalea psychrophila LSv54 uid58153* | DP2165 | L,L-diaminopimelate aminotransferase |
| *Desulfotomaculum acetoxidans DSM 771 uid59109* | Dtox_2324 | L,L-diaminopimelate aminotransferase |
| *Desulfotomaculum carboxydivorans CO 1 SRB uid67317* | Desca_1275 | L,L-diaminopimelate aminotransferase |
| *Desulfotomaculum gibsoniae DSM 7213 uid76945* | Desgi_3001 | L,L-diaminopimelate aminotransferase |
| *Desulfotomaculum kuznetsovii DSM 6115 uid67357* | Desku_1301 | L,L-diaminopimelate aminotransferase |
| *Desulfotomaculum reducens MI 1 uid58277* | Dred_1695 | L,L-diaminopimelate aminotransferase |
| *Desulfotomaculum ruminis DSM 2154 uid67507* | Desru_2323 | L,L-diaminopimelate aminotransferase |
| *Desulfovibrio africanus Walvis Bay uid66847* | Desaf_3033 | L,L-diaminopimelate aminotransferase |
| *Desulfovibrio alaskensis G20 uid57941* | Dde_1970 | L,L-diaminopimelate aminotransferase |
| *Desulfovibrio desulfuricans ATCC 27774 uid59213* | Ddes_1151 | class I and II aminotransferase |
| *Desulfovibrio gigas DSM 1382 uid221293* | DGI_2278 | Putative L,L-diaminopimelate aminotransferase |
| *Desulfovibrio hydrothermalis AM13 DSM 14728 uid184831* | DESAM_22304 | L,L-diaminopimelate aminotransferase |
| *Desulfovibrio hydrothermalis AM13 DSM 14728 uid184831* | DESAM_22304 | L,L-diaminopimelate aminotransferase |
| *Desulfovibrio piezophilus C1TLV30 uid190704* | BN4_10702 | L,L-diaminopimelate aminotransferase |
| *Desulfovibrio salexigens DSM 2638 uid59223* | Desal_1952 | L,L-diaminopimelate aminotransferase |
| *Desulfovibrio vulgaris Miyazaki F uid59089* | DvMF_0361 | L,L-diaminopimelate aminotransferase |
| *Desulfovibrio vulgaris DP4 uid58679* | Dvul_1431 | L,L-diaminopimelate aminotransferase |
| *Desulfovibrio vulgaris Hildenborough uid57645* | DVU1655 | L,L-diaminopimelate aminotransferase |
| *Desulfovibrio vulgaris RCH1 uid161961* | Deval_1636 | L,L-diaminopimelate aminotransferase |
| *Desulfurivibrio alkaliphilus AHT2 uid49487* | DaAHT2_2137 | L,L-diaminopimelate aminotransferase |
| *Eggerthella YY7918 uid68707* | EGYY_20270 | PLP-dependent aminotransferase |
| *Elusimicrobium minutum Pei191 uid58949* | Emin_1014 | L,L-diaminopimelate aminotransferase |
| *Ethanoligenens harbinense YUAN 3 uid46255* | Ethha_0920 | L,L-diaminopimelate aminotransferase |
| *Eubacterium eligens ATCC 27750 uid59171* | EUBELI_00672 | L,L-diaminopimelate aminotransferase |
| *Eubacterium rectale ATCC 33656 uid59169* | EUBREC_2541 | L,L-diaminopimelate aminotransferase |
| *Eubacterium rectale uid197161* | EUR_26020 | L,L-diaminopimelate aminotransferase apoenzyme |
| *Eubacterium rectale uid197162* | ERE_08100 | L,L-diaminopimelate aminotransferase apoenzyme |
| *Eubacterium siraeum uid197160* | EUS_01380 | L,L-diaminopimelate aminotransferase apoenzyme |
| *Eubacterium siraeum V10Sc8a uid197178* | ES1_03640 | L,L-diaminopimelate aminotransferase apoenzyme |
| *Faecalibacterium prausnitzii L2 6 uid197183* | FP2_15790 | L,L-diaminopimelate aminotransferase apoenzyme |
| *Faecalibacterium prausnitzii uid197157* | FPR_20330 | L,L-diaminopimelate aminotransferase apoenzyme |
| *Fibrobacter succinogenes S85 uid161919* | FSU_2316 | class I/II aminotransferase |
| *Fibrobacter succinogenes S85 uid41169* | Fisuc_1814 | L,L-diaminopimelate aminotransferase |
| *gamma proteobacterium HdN1 uid51635* | HDN1F_30690 | aminotransferase |
| *Geitlerinema PCC 7407 uid183007* | GEI7407_1967 | L,L-diaminopimelate aminotransferase apoenzyme |
| *Geobacter bemidjiensis Bem uid58749* | Gbem_4052 | L,L-diaminopimelate aminotransferase |
| *Geobacter FRC 32 uid58543* | Geob_1134 | L,L-diaminopimelate aminotransferase |
| *Geobacter lovleyi SZ uid58713* | Glov_3040 | L,L-diaminopimelate aminotransferase |
| *Geobacter M18 uid55771* | GM18_4513 | L,L-diaminopimelate aminotransferase |
| *Geobacter M21 uid59037* | GM21_4142 | L,L-diaminopimelate aminotransferase |
| *Geobacter metallireducens GS 15 uid57731* | Gmet_0213 | L,L-diaminopimelate aminotransferase |
| *Geobacter sulfurreducens KN400 uid161977* | KN400_0137 | L,L-diaminopimelate aminotransferase |
| *Geobacter sulfurreducens PCA uid57743* | GSU0162 | L,L-diaminopimelate aminotransferase |
| *Geobacter uraniireducens Rf4 uid58475* | Gura_0238 | L,L-diaminopimelate aminotransferase |
| *Gloeobacter JS uid225602* | GKIL_3332 | L,L-diaminopimelate aminotransferase |
| *Gloeobacter violaceus PCC 7421 uid58011* | glr4108 | L,L-diaminopimelate aminotransferase |
| *Gloeocapsa PCC 7428 uid183112* | Glo7428_2036 | L,L-diaminopimelate aminotransferase apoenzyme |
| *Halothece PCC 7418 uid183338* | PCC7418_2518 | L,L-diaminopimelate aminotransferase apoenzyme |
| *Halothermothrix orenii H 168 uid58585* | Hore_11560 | L,L-diaminopimelate aminotransferase |
| *Heliobacterium modesticaldum Ice1 uid58279* | HM1_0993 | L,L-diaminopimelate aminotransferase |
| *Hydrogenobacter thermophilus TK 6 uid159875* | Hydth_0355 | L,L-diaminopimelate aminotransferase |
| *Hydrogenobacter thermophilus TK 6 uid45927* | HTH_0357 | aminotransferase |
| *Hydrogenobaculum HO uid190882* | HydHO_0476 | L,L-diaminopimelate aminotransferase apoenzyme |
| *Hydrogenobaculum SN uid46251* | HydSN_0485 | L,L-diaminopimelate aminotransferase apoenzyme |
| *Hydrogenobaculum Y04AAS1 uid58857* | HY04AAS1_0487 | L,L-diaminopimelate aminotransferase |
| *Kytococcus sedentarius DSM 20547 uid59071* | Ksed_00770 | L,L-diaminopimelate aminotransferase |
| *Lawsonia intracellularis N343 uid186598* | LAW_00450 | L,L-diaminopimelate aminotransferase |
| *Lawsonia intracellularis PHE MN1 00 uid61575* | LI0435 | L,L-diaminopimelate aminotransferase |
| *Leptolyngbya PCC 7376 uid182928* | Lepto7376_0699 | L,L-diaminopimelate aminotransferase apoenzyme |
| *Leptospira biflexa serovar Patoc Patoc 1 Ames uid58511* | LBF_0994 | L,L-diaminopimelate aminotransferase |
| *Leptospira biflexa serovar Patoc Patoc 1 Paris uid58993* | LEPBI_I1028 | L,L-diaminopimelate aminotransferase |
| *Leptospira borgpetersenii serovar Hardjo bovis JB197 uid58509* | LBJ_0733 | L,L-diaminopimelate aminotransferase |
| *Leptospira borgpetersenii serovar Hardjo bovis L550 uid58507* | LBL_2347 | L,L-diaminopimelate aminotransferase |
| *Leptospira interrogans serovar Copenhageni Fiocruz L1 130 uid58065* | LIC12841 | L,L-diaminopimelate aminotransferase |
| *Leptospira interrogans serovar Lai 56601 uid57881* | LA_0776 | L,L-diaminopimelate aminotransferase |
| *Leptospira interrogans serovar Lai IPAV uid161957* | LIF_A0633 | L,L-diaminopimelate aminotransferase |
| *Megamonas hypermegale uid197163* | MHY_15860 | L,L-diaminopimelate aminotransferase apoenzyme |
| *Megasphaera elsdenii DSM 20460 uid71135* | MELS_0042 | L,L-diaminopimelate aminotransferase |
| *Methanobacterium AL 21 uid63623* | Metbo_0824 | L,L-diaminopimelate aminotransferase |
| *Methanobacterium MB1 uid231690* | MBMB1_0946 | L,L-diaminopimelate aminotransferase |
| *Methanobacterium SWAN 1 uid67359* | MSWAN_1421 | L,L-diaminopimelate aminotransferase |
| *Methanobrevibacter AbM4 uid206516* | Abm4_1220 | diaminopimelate aminotransferase DapL |
| *Methanobrevibacter ruminantium M1 uid45857* | mru_0941 | diaminopimelate aminotransferase DapL |
| *Methanobrevibacter smithii ATCC 35061 uid58827* | Msm_1455 | L,L-diaminopimelate aminotransferase |
| *Methanosphaera stadtmanae DSM 3091 uid58407* | Msp_0924 | L,L-diaminopimelate aminotransferase |
| *Methanothermobacter marburgensis Marburg uid51637* | MTBMA_c05430 | L,L-diaminopimelate aminotransferase |
| *Methanothermobacter thermautotrophicus Delta H uid57877* | MTH52 | L,L-diaminopimelate aminotransferase |
| *Methanothermus fervidus DSM 2088 uid60167* | Mfer_0901 | L,L-diaminopimelate aminotransferase apoenzyme |
| *Microcoleus PCC 7113 uid183114* | Mic7113_4760 | L,L-diaminopimelate aminotransferase apoenzyme |
| *Microcystis aeruginosa NIES 843 uid59101* | MAE_46460 | L,L-diaminopimelate aminotransferase |
| *Moorella thermoacetica ATCC 39073 uid58051* | Moth_0889 | L,L-diaminopimelate aminotransferase |
| *Natranaerobius thermophilus JW NM WN LF uid59001* | Nther_1272 | class I and II aminotransferase |
| *Nostoc azollae 0708 uid49725* | Aazo_1424 | L,L-diaminopimelate aminotransferase |
| *Nostoc PCC 7107 uid182932* | Nos7107_2694 | L,L-diaminopimelate aminotransferase apoenzyme |
| *Nostoc PCC 7107 uid182932* | Nos7107_3901 | L,L-diaminopimelate aminotransferase apoenzyme |
| *Nostoc PCC 7120 uid57803* | all4327 | L,L-diaminopimelate aminotransferase |
| *Nostoc PCC 7120 uid57803* | alr5103 | L,L-diaminopimelate aminotransferase |
| *Nostoc PCC 7524 uid182933* | Nos7524_3268 | L,L-diaminopimelate aminotransferase apoenzyme |
| *Nostoc PCC 7524 uid182933* | Nos7524_4239 | Aspartate/tyrosine/aromatic aminotransferase |
| *Nostoc punctiforme PCC 73102 uid57767* | Npun_R3615 | L,L-diaminopimelate aminotransferase |
| *Opitutus terrae PB90 1 uid58965* | Oter_4620 | L,L-diaminopimelate aminotransferase |
| *Oscillatoria acuminata PCC 6304 uid183003* | Oscil6304_3181 | L,L-diaminopimelate aminotransferase apoenzyme |
| *Oscillatoria acuminata PCC 6304 uid183003* | Oscil6304_4156 | Aspartate/tyrosine/aromatic aminotransferase |
| *Oscillatoria PCC 7112 uid183110* | Osc7112_3351 | L,L-diaminopimelate aminotransferase apoenzyme |
| *Paludibacter propionicigenes WB4 uid60725* | Palpr_0801 | L,L-diaminopimelate aminotransferase apoenzyme |
| *Paludibacter propionicigenes WB4 uid60725* | Palpr_1095 | L,L-diaminopimelate aminotransferase apoenzyme |
| *Parabacteroides distasonis ATCC 8503 uid58301* | BDI_0321 | L,L-diaminopimelate aminotransferase |
| *Parachlamydia acanthamoebae UV7 uid68335* | PUV_02300 | L,L-diaminopimelate aminotransferase |
| *Pelobacter carbinolicus DSM 2380 uid58241* | Pcar_2423 | L,L-diaminopimelate aminotransferase |
| *Pelobacter propionicus DSM 2379 uid58255* | Ppro_3061 | L,L-diaminopimelate aminotransferase |
| *Pelotomaculum thermopropionicum SI uid58877* | PTH_1799 | L,L-diaminopimelate aminotransferase |
| *Phycisphaera mikurensis NBRC 102666 uid157331* | PSMK_11600 | L,L-diaminopimelate aminotransferase |
| *Planctomyces brasiliensis DSM 5305 uid60583* | Plabr_2004 | L,L-diaminopimelate aminotransferase apoenzyme |
| *Planctomyces limnophilus DSM 3776 uid48643* | Plim_3995 | class I and II aminotransferase |
| *Pleurocapsa PCC 7327 uid183006* | Ple7327_4196 | L,L-diaminopimelate aminotransferase |
| *Prevotella ruminicola 23 uid47507* | PRU_1974 | aminotransferase-like protein |
| *Prochlorococcus marinus AS9601 uid58307* | A9601_17031 | L,L-diaminopimelate aminotransferase |
| *Prochlorococcus marinus CCMP1375 uid57995* | Pro_1655 | Aspartate aminotransferase family enzyme |
| *Prochlorococcus marinus MIT 9211 uid58309* | P9211_16221 | L,L-diaminopimelate aminotransferase |
| *Prochlorococcus marinus MIT 9215 uid58819* | P9215_17681 | L,L-diaminopimelate aminotransferase |
| *Prochlorococcus marinus MIT 9301 uid58437* | P9301_16911 | L,L-diaminopimelate aminotransferase |
| *Prochlorococcus marinus MIT 9303 uid58305* | P9303_23741 | L,L-diaminopimelate aminotransferase |
| *Prochlorococcus marinus MIT 9312 uid58357* | PMT9312_1592 | L,L-diaminopimelate aminotransferase |
| *Prochlorococcus marinus MIT 9313 uid57773* | PMT1791 | L,L-diaminopimelate aminotransferase |
| *Prochlorococcus marinus MIT 9515 uid58313* | P9515_16801 | L,L-diaminopimelate aminotransferase |
| *Prochlorococcus marinus NATL1A uid58423* | NATL1_19411 | L,L-diaminopimelate aminotransferase |
| *Prochlorococcus marinus NATL2A uid58359* | PMN2A_1066 | L,L-diaminopimelate aminotransferase |
| *Prochlorococcus marinus pastoris CCMP1986 uid57761* | PMM1500 | L,L-diaminopimelate aminotransferase |
| *Pseudanabaena PCC 7367 uid183004* | Pse7367_1111 | L,L-diaminopimelate aminotransferase apoenzyme |
| *Rivularia PCC 7116 uid182929* | Riv7116_5758 | L,L-diaminopimelate aminotransferase apoenzyme |
| *Roseburia hominis A2 183 uid73419* | RHOM_05295 | L,L-diaminopimelate aminotransferase |
| *Roseburia intestinalis uid197164* | ROI_10700 | L,L-diaminopimelate aminotransferase apoenzyme |
| *Roseburia intestinalis XB6B4 uid197179* | RO1_22920 | L,L-diaminopimelate aminotransferase apoenzyme |
| *Ruminococcus obeum uid197165* | CK5_31360 | L,L-diaminopimelate aminotransferase apoenzyme |
| *Ruminococcus albus 7 uid51721* | Rumal_1487 | L,L-diaminopimelate aminotransferase |
| *Ruminococcus bromii uid197158* | RBR_01390 | L,L-diaminopimelate aminotransferase apoenzyme |
| *Ruminococcus champanellensis 18P13 uid197169* | RUM_18400 | L,L-diaminopimelate aminotransferase apoenzyme |
| *Ruminococcus uid197156* | CK1_18280 | L,L-diaminopimelate aminotransferase apoenzyme |
| *Selenomonas ruminantium lactilytica TAM6421 uid157247* | SELR_16200 | Putative L,L-diaminopimelate aminotransferase |
| *Selenomonas sputigena ATCC 35185 uid55329* | Selsp_1616 | L,L-diaminopimelate aminotransferase |
| *Simkania negevensis Z uid68451* | SNE_A10370 | L,L-diaminopimelate aminotransferase |
| *Sphaerochaeta pleomorpha Grapes uid82365* | SpiGrapes_0767 | L,L-diaminopimelate aminotransferase |
| *Spirochaeta africana DSM 8902 uid81779* | Spiaf_1724 | L,L-diaminopimelate aminotransferase |
| *Spirochaeta Buddy uid63633* | SpiBuddy_1123 | L,L-diaminopimelate aminotransferase |
| *Spirochaeta caldaria DSM 7334 uid68753* | Spica_0952 | L,L-diaminopimelate aminotransferase |
| *Spirochaeta coccoides DSM 17374 uid66331* | Spico_0778 | L,L-diaminopimelate aminotransferase apoenzyme |
| *Spirochaeta L21 RPul D2 uid231658* | L21SP2_1960 | L,L-diaminopimelate aminotransferase |
| *Spirochaeta smaragdinae DSM 11293 uid51369* | Spirs_1461 | L,L-diaminopimelate aminotransferase |
| *Spirochaeta thermophila DSM 6192 uid53037* | STHERM_c17630 | aminotransferase |
| *Spirochaeta thermophila DSM 6578 uid162041* | Spith_1834 | L,L-diaminopimelate aminotransferase |
| *Stanieria cyanosphaera PCC 7437 uid183115* | Sta7437_2464 | L,L-diaminopimelate aminotransferase apoenzyme |
| *Synechococcus CC9311 uid58123* | sync_0372 | L,L-diaminopimelate aminotransferase |
| *Synechococcus CC9605 uid58319* | Syncc9605_0311 | L,L-diaminopimelate aminotransferase |
| *Synechococcus CC9902 uid58323* | Syncc9902_2031 | L,L-diaminopimelate aminotransferase |
| *Synechococcus elongatus PCC 6301 uid58235* | syc0687_c | L,L-diaminopimelate aminotransferase |
| *Synechococcus elongatus PCC 7942 uid58045* | Synpcc7942_0853 | L,L-diaminopimelate aminotransferase |
| *Synechococcus JA 2 3B a 2 13 uid58537* | CYB_1421 | L,L-diaminopimelate aminotransferase |
| *Synechococcus JA 3 3Ab uid58535* | CYA_2460 | L,L-diaminopimelate aminotransferase |
| *Synechococcus PCC 6312 uid182934* | Syn6312_3115 | L,L-diaminopimelate aminotransferase apoenzyme |
| *Synechococcus PCC 7002 uid59137* | SYNPCC7002_A1203 | L,L-diaminopimelate aminotransferase |
| *Synechococcus PCC 7502 uid183008* | Syn7502_03457 | L,L-diaminopimelate aminotransferase apoenzyme |
| *Synechococcus RCC307 uid61609* | SynRCC307_2179 | L,L-diaminopimelate aminotransferase |
| *Synechococcus WH 7803 uid61607* | SynWH7803_0370 | L,L-diaminopimelate aminotransferase |
| *Synechococcus WH 8102 uid61581* | SYNW2147 | L,L-diaminopimelate aminotransferase |
| *Synechocystis PCC 6803 substr GT I uid157913* | SYNGTI_2620 | hypothetical protein |
| *Synechocystis PCC 6803 substr GT I uid158059* | SYNPCCN_2619 | hypothetical protein |
| *Synechocystis PCC 6803 substr PCC N uid159835* | SYNPCCP_2619 | hypothetical protein |
| *Synechocystis PCC 6803 uid159873* | SYNGTS_2621 | hypothetical protein |
| *Synechocystis PCC 6803 uid189748* | MYO_126460 | hypothetical protein |
| *Synechocystis PCC 6803 uid57659* | sll0480 | L,L-diaminopimelate aminotransferase |
| *Syntrophobacter fumaroxidans MPOB uid58177* | Sfum_0054 | class I/II aminotransferase |
| *Syntrophobotulus glycolicus DSM 8271 uid63343* | Sgly_2396 | L,L-diaminopimelate aminotransferase apoenzyme |
| *Syntrophothermus lipocalidus DSM 12680 uid49527* | Slip_0851 | L,L-diaminopimelate aminotransferase |
| *Thermacetogenium phaeum DSM 12270 uid177811* | Tph_c14370 | L,L-diaminopimelate aminotransferase DapL |
| *Thermincola potens JR uid48823* | TherJR_1813 | L,L-diaminopimelate aminotransferase |
| *Thermobacillus composti KWC4 uid74021* | Theco_2294 | L,L-diaminopimelate aminotransferase |
| *Thermocrinis albus DSM 14484 uid46231* | Thal_1518 | L,L-diaminopimelate aminotransferase |
| *Thermodesulfatator indicus DSM 15286 uid68285* | Thein_0755 | L,L-diaminopimelate aminotransferase |
| *Thermosediminibacter oceani DSM 16646 uid51421* | Toce_1019 | L,L-diaminopimelate aminotransferase apoenzyme |
| *Thermosynechococcus elongatus BP 1 uid57907* | tll2102 | L,L-diaminopimelate aminotransferase |
| *Thermosynechococcus NK55 uid231517* | NK55_08525 | L,L-diaminopimelate aminotransferase DapL |
| *Treponema azotonutricium ZAS 9 uid67365* | TREAZ_1309 | L,L-diaminopimelate aminotransferase |
| *Treponema brennaborense DSM 12168 uid66607* | Trebr_0759 | L,L-diaminopimelate aminotransferase |
| *Treponema primitia ZAS 2 uid67367* | TREPR_1185 | L,L-diaminopimelate aminotransferase |
| *Treponema succinifaciens DSM 2489 uid65781* | Tresu_0418 | L,L-diaminopimelate aminotransferase |
| *Trichodesmium erythraeum IMS101 uid57925* | Tery_3293 | L,L-diaminopimelate aminotransferase |
| *Veillonella parvula DSM 2008 uid41927* | Vpar_0331 | class I and II aminotransferase |
| *Waddlia chondrophila WSU 86 1044 uid49531* | wcw_0764 | L,L-diaminopimelate aminotransferase |
